# Survival and reduction in foodborne bacteria using methyl cellulose film doped with europium oxide nanoparticles

**DOI:** 10.1002/fsn3.1305

**Published:** 2019-12-04

**Authors:** Mahmoud Fawzy Abd Elkader, Mohammed Suliman Almogbel, Mohamed Tharwat Elabbasy

**Affiliations:** ^1^ Biophysics Department Faculty of Science Cairo University Giza Egypt; ^2^ Clinical laboratory sciences Department College of Applied Medical Sciences Ha'il University Ha'il Saudi Arabia; ^3^ Molecular Diagnostics and Personalised Therapeutics Unit (MDPTU) Ha'il University Ha'il Saudi Arabia; ^4^ Public Health Department College of Public Health and Health Informatics Ha'il University Ha'il Saudi Arabia; ^5^ Food Control Department Faculty of Veterinary Medicine Zagazig University Zagazig Egypt

**Keywords:** Eu_2_O_3_ nanoparticles, food packaging and antimicrobial activity, foodborne bacteria, methyl cellulose

## Abstract

The study validated the efficacy of methyl cellulose films doped with different concentration of Eu_2_O_3_ nanoparticles to inactivate foodborne pathogens. Eu_2_O_3_ nanoparticles were added to the methyl cellulose solution with different weight percentages (0.0, 0.5, 0.75, 1.0, 1.25, and 1.5 wt%). X‐ray diffraction patterns for the prepared films were studied. A significant lower count of *E. coli, S. typhimurium,* and *S. aureus* (*p* ≤ .05) inoculated in MC films doped with Eu_2_O_3_ nanoparticles compared with pure MC film could be achieved. The findings acquired verify the impact of prepared MC films doped with Eu_2_O_3_ nanoparticles on the test strains.

## Graphical abstract

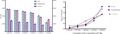

## 1 INTRODUCTION

An escalation has been found in foodborne outbreaks caused by pathogens especially in developing countries. These foodborne outbreaks cause most of food contamination and poisoning cases leading to serious diseases and may be to death (Blackburn & McClure, 2009; Chen, Tang, Liu, Cai, & Bai, 2012; Crim et al., 2015; Organization, 2012; Team, 2013). Foodborne bacteria may contaminate food by nonfood mechanisms and represent a potential public health threat (Chen et al., 2012; Scallan et al., 2011). *E. coli, S. typhimurium,* and *S. aureus* remain a significant food safety issue in raw meat, chicken meat, and their products (Chen et al., 2012). Despite the introduction of mandatory testing for foodborne bacteria and multi‐level intervention strategies, sporadic outbreaks of foodborne diseases and products recall are associated with *E. coli, S. typhimurium*, and *S. aureus* contamination (Wadamori, Gooneratne, & Hussain, 2017)*.*

*E. coli* has turned into an expanding worry to the meat industry and public health (Gorton & Stasiewicz, 2017)*. S. typhimurium* infections pose significant public health globally. *S. typhimurium* are most common causes of foodborne illness in humans (Jain et al., 2009). *S. typhimurium* usually spread through inappropriately handled food that has come in contact with animal or human feces and they are responsible for the majority of foodborne illnesses (de Freitas et al., 2010). *S. aureus* strains have been demonstrated as one of the world's significant causes of foodborne diseases (Balaban & Rasooly, 2000; Bianchi et al., 2014).

As a consequence, there is a need for a developed packaging material to be used in meat and poultry industry that have the ability to reduce the carriage of foodborne pathogens, and to be used with other decontamination strategies to achieve satisfactory safety rates.

Developing of naturally occurring polymer with the film formation capacity and antimicrobial properties to improve health, safety, shelf life, and biomedical application, gains a considerable regard nowadays (Fernandez‐Saiz, Lagaron, Hernandez‐Muñoz, & Ocio, 2008; Irkin & Esmer, 2015; Malhotra, Keshwani, & Kharkwal, 2015). Despite the common use of cellulose and its derivatives in food packaging, researchers look to improve its antimicrobial properties in the future. Cellulose is one in all the foremost various and biodegradable compound that insoluble in water and most organic solvent (Coffey, Bell, & Henderson, 1995; El‐Kader & Ragab, 2013). Substitution of hydroxyl groups within the backbone of cellulose by some functional groups makes it water soluble such as methyl groups in methyl cellulose (MC). Methyl cellulose has an excellent film formation capacity, water solubility, and efficient oxygen and lipid permeability (Chevillard & Axelos, 1997; Nasatto et al., 2015). In literature, there are various studies about preparation of functional methyl cellulose nanocomposite films and their physicochemical (Tunç & Duman, 2010), antibacterial (Tunç & Duman, 2011), mechanical, and gas barrier (Tunc, Duman, & Polat, 2016) properties.

A great interest for biomedical applications has been emphasized to develop biopolymers by adding nanoparticles to its matrix. Doping biopolymers with nanoparticles is the concern of the research and industrial world, as they exhibit physical, chemical and antimicrobial enhancement (Carbone, Donia, Sabbatella, & Antiochia, 2016; Li, He, Li, & Zhang, 2015; Muthulakshmi, Rajini, Rajalu, Siengchin, & Kathiresan, 2017). The use of rare earth elements nanoparticles as a dopant for different biopolymers can be considered as a way to develop bilateral.

Among the rare earth elements, Eu(III) was the focus of many studies. It has a wide applications ranging from telecommunications to biomedical applications (Diallo, Mothudi, Manikandan, & Maaza, 2016; Feng & Zhang, 2013; Mahajan & Dickerson, 2010; Quesada, del Campo, & Fernández, 2015). Europium(III) Oxide (Eu_2_O_3_) is the simplest oxide of Eu(III) element, and it was obtained from the thermal annealing of Eu hydroxide under high temperatures(Kang, Jung, Min, & Sohn, 2014).

In our study, we will use Eu_2_O_3_ nanoparticles as a dopant in the MC matrix with different concentrations to form a thin films, which were then defined by x‐ray diffraction process. The prepared MC films were examined to evaluate their consequences on the used foodborne test strains.

## 2 MATERIALS AND METHODS

### 2.1 Preparation of MC films doped with Eu_2_O_3_ nanoparticles

MC of 2% aqueous solution at 20°C was provided by LOBA Chemie, India, with viscosity of 350–550 cP and pH values of 5.5–8.0. Eu_2_O_3_ nanoparticles was supplied by Sigma‐Aldrich, its density was 7.42 g/ml at 20°C, and the particle size was less than 150 nm. All glasswares were thoroughly cleaned in aqua region and rinsed copiously with double distilled water.

To prepare thin film of MC, incorporated with Eu_2_O_3_ nanoparticles, two grams of MC were dissolved in 100 ml double distilled water at 50°C using a magnetic stirrer overnight. Eu_2_O_3_ nanoparticles were added to the MC solution with different weight percentages (0.00, 0.50, 0.75, 1.00, 1.25, and 1.50 wt%) and were stirred for 12 hr at 50°C. The solution was cast in stainless‐steel plates with diameter 12 cm and then dried in open air at room temperature for 3 days until solvent was nearly evaporated. The obtained films were of suitable thickness ≈ 100μm.

### 2.2 X‐ray diffraction

The amorphous/crystalline nature of methyl cellulose/Eu_2_O_3_ nanocomposite films was checked by using DIANO X‐ray defractometer equipped with Cu‐K_α_ radiation (λ = 1.54056 A^o^, operation voltage = 30 kV).

### 2.3 Antimicrobial activity

#### 2.3.1 Preparation of test strains

*E. coli, S. typhimurium,* and *S. aureus* were obtained from the Microbiology laboratory of Molecular Diagnostic and Personalised Therapeutics unit (MDXPTU), Hail University. Strains were originally isolated from chicken meat samples, then were identified biochemically, serologically, phenotypically, and genotypically. Strains were saved in the MDXPTU Biobank at −80°C. Each strain was cultivated separately in Tryptic soy broth (Difco) at 37°C for 24 hr. The cells were harvested by centrifugation 5,000 *g*/10 min and were washed twice then were resuspended to a final cell density of 7 log cfu/ml (OD_600_ 0.2) using sterile saline (0.85% NaCl).

#### 2.3.2 Bacterial inoculation

MC films doped with different concentration of Eu_2_O_3_ nanoparticles were tested antimicrobially against MC control film. All the tested MC films were cut aseptically to form an area of 1 cm^2^, each area was inoculated with 10 µl of test strains (8 log cfu/ml concentrations) (*E. coli, S. typhimurium and S. aureus*). The inoculated MC films were kept in bio‐safety cabinet to dry for 2 hr.

#### 2.3.3 Survival and reduction in test strains

The survival and reduction in the different test strains inoculated on the MC films doped with different concentration of Eu_2_O_3_ nanoparticles were determined against the pure MC film. About 10 ml of sterile phosphate‐buffered saline was added to the Inoculated MC films (area of 1 cm^2^) in sterile tubes. Vigorous shaking was carried out to the tubes using vortex for 3 min. A 10‐fold serial dilution was prepared. Dilutions were plated in duplicate onto Tryptic Soya agar and Mueller‐Hinton agar (Difco) that was subsequently incubated at 37°C for 24 hr.

#### 2.3.4 Bacterial adherence assay

A laboratory‐based trials were undertaken to determine the binding strength of the different tested serotypes to the MC films doped with different concentration of Eu_2_O_3_ nanoparticles against the pure MC film. The Initial adhesion assays to MC films doped with different concentration of Eu_2_O_3_ nanoparticles were determined. About 10 µl of the test strains (8 log cfu/ml concentrations) were added to MC films doped with different concentration of Eu_2_O_3_ nanoparticles (with an area of 1 cm^2^). Three times of rinsing were performed after 30 min of adhesion by utilizing the phosphate‐buffered saline. Numbers recovered were used to estimate the weak attachment strength of the bacterial cells to the film surface. The washed film was vigorously shaken using vortex for 5 min, and the levels recovered in the homogenate used to estimate the strongly attached portion of the population.

#### 2.3.5 Pulse field gel electrophoresis (PFGE)

PFGE was conducted to identify the clonal relatedness of the test strains after inoculation on MC films. Already optimized protocol following Standard Operating Procedure (SOP) for PulseNet PFGE using a CHEF‐Mapper (Bio‐Rad Laboratories) was used.

### 2.4 Statistical analysis

The mean values with standard error of the means (*SEM*) were calculated. One way analysis of variance (ANOVA) at 95% level of confidence and least Significant difference (LSD) post hoc were done to determine significant differences (*p* < .05 was considered as significant).

## 3 RESULT AND DISCUSSION

### 3.1 X‐ray diffraction (XRD)

**Figure 1:**
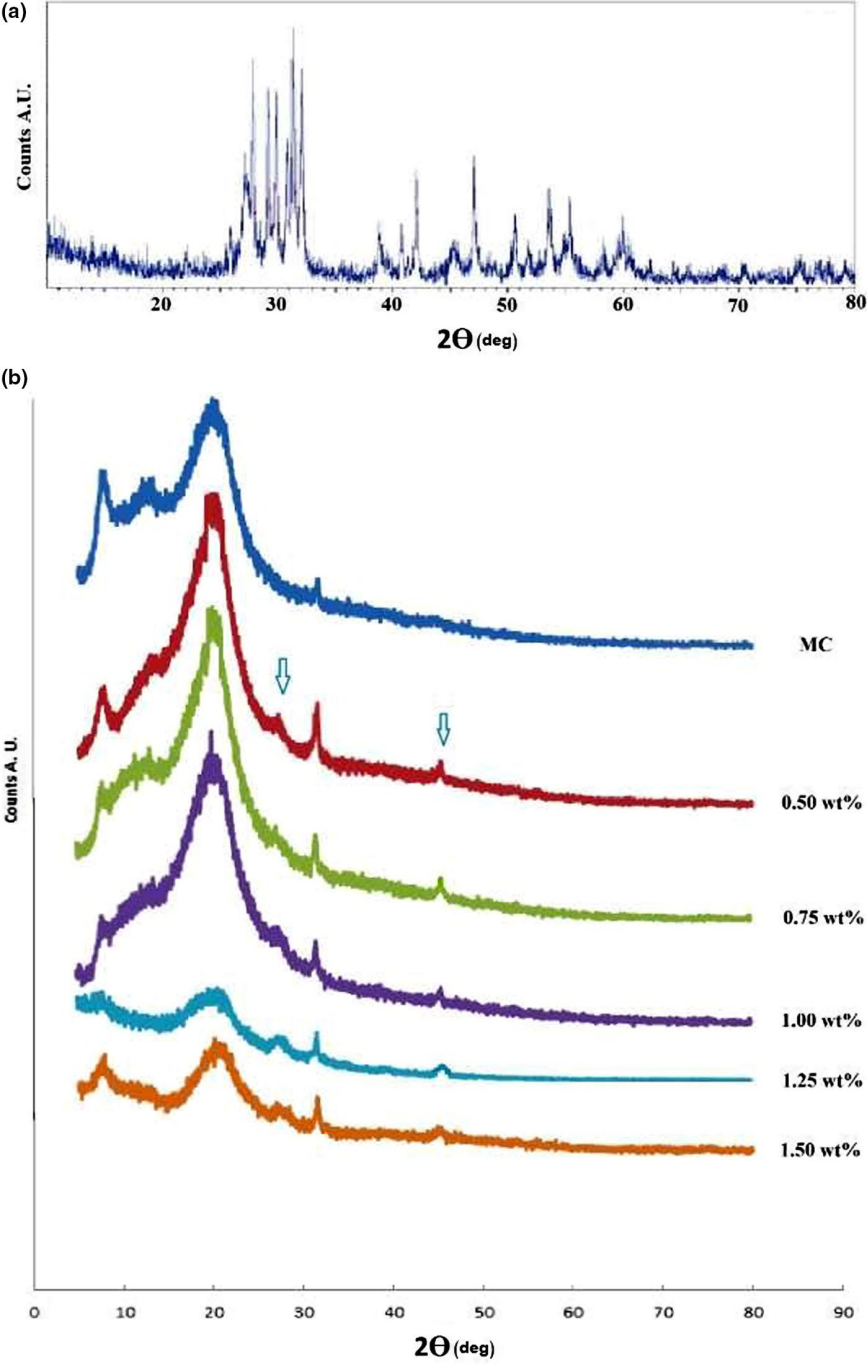
X‐ray diffraction patterns of (a) pure Eu_2_O_3_ nanoparticles, (b) MC film doped with different concentration of Eu_2_O_3_ nanoparticles

Figure 1 present X‐ray diffraction patterns of pure Eu_2_O_3_ nanoparticles (Figure 1a) and of MC/Eu_2_O_3_ nanocomposite films (Figure 1b).

The X‐ray diffraction pattern of pure Eu_2_O_3_ nanoparticles (Figure 1a) revealed sharp crystalline peaks appeared at 28°, 29.5°, 31.5°, 39°, 42°, 45.5°, 47°, 51°, 54°, 55°, 58°, and 59.5° corresponding to high crystallized form of the cubic Eu_2_O_3_ nanoparticles (Kang et al., 2014).

The XRD pattern of the MC homopolymer shows a semicrystalline structure revels three peaks (Figure 1b), a sharp one at 2ϴ = 8° corresponds to the trimethylglucose‐type crystalline order (Kato, Yokoyama, & Takahashi, 1978). A broad peak with maximum at 2ϴ = 21° indicates the intermolecular structure of MC (Rangelova et al., 2011), and a weak peak appeared at 2ϴ = 13.3° which indicates a more hydrated structure (Liebeck, Hidalgo, Roth, Popescu, & Böker, 2017).

The XRD patterns of MC/Eu_2_O_3_ nanocomposites (Figure 1b) exhibits the characteristic features of the pure MC homopolymer, but with less intensity of the reflection peak at 2ϴ = 8°. The reflection peak intensity at 2ϴ = 21° for MC films doped with 0.50, 0.75, and 1.00 wt%. Eu_2_O_3_ nanoparticles was significantly increased, this can be due to the higher oxidation number in the Eu (III) oxide, leading to forming new bonds. A faint crystalline peaks appeared at 2ϴ = 28° and 46° in all MC films doped with Eu_2_O_3_ nanoparticles, and this can be attributed to the incorporation of Eu_2_O_3_ nanoparticles into the polymer matrix.

The degree of crystallinity of MC films doped with different concentration of Eu_2_O_3_ nanoparticles (0.00, 0.50, 0.75, 1.00, 1.25, 1.50 wt%) was calculated using the Hermans‐Weidinger method (Hermans & Weidinger, 1961), and it was determined to be 17.8, 28.5, 31.0, 25.2, 20.4, and 21.9, respectively.

The decrease in the degree of crystallinity of MC films doped with 1.25, and 1.50 wt% Eu_2_O_3_ NPs was due to the saturation effect. From the data on the degree of crystallinity, it was noticed that the values of the degree of crystallinity for the composite samples are higher than that of the MC homopolymer.

### 3.2 Antimicrobial activity

**Figure 2:**
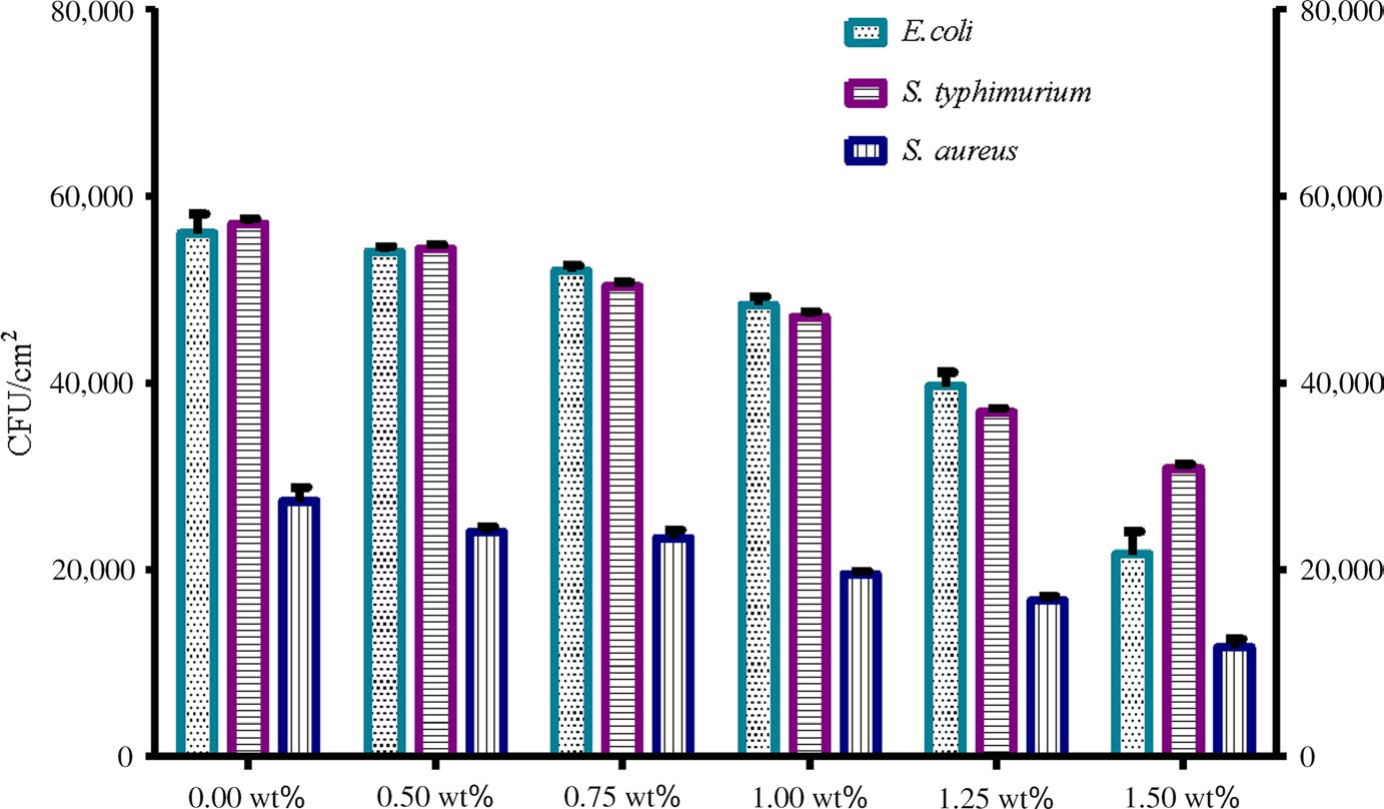
Effect of MC films doped with different concentration of Eu_2_O_3_ nanoparticles on viable count of test strains

**Figure 3:**
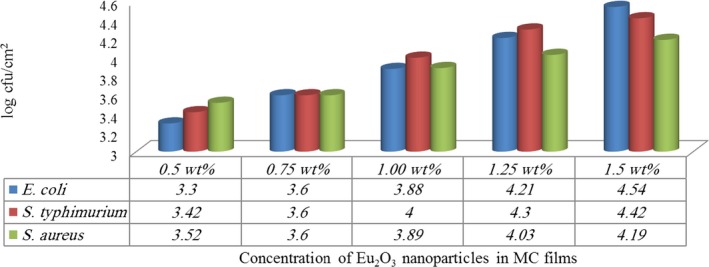
Log reduction in test strains in MC films doped with different concentration of Eu_2_O_3_ nanoparticles

The obtained results in this study revealed that MC films doped with different concentration of Eu_2_O_3_ nanoparticles have been shown to possess potential antibacterial activity against the used foodborne test strains that previously isolated from chicken meat products. A significant lower count of *E. coli, S. typhimurium,* and *S. aureus* (*p* ≤ .05) inoculated in MC films doped with different concentration of Eu_2_O_3_ nanoparticles compared to pure MC film could confirm the effect of the prepared films against foodborne test strains (Figures 2 and 3). MC films doped with 1.50 wt% Eu_2_O_3_ nanoparticles exhibited the strongest activity against the test strains, while MC films doped with 0.50 wt% Eu_2_O_3_ nanoparticles were the least effective concentration compared with pure MC film.

**Figure 4:**
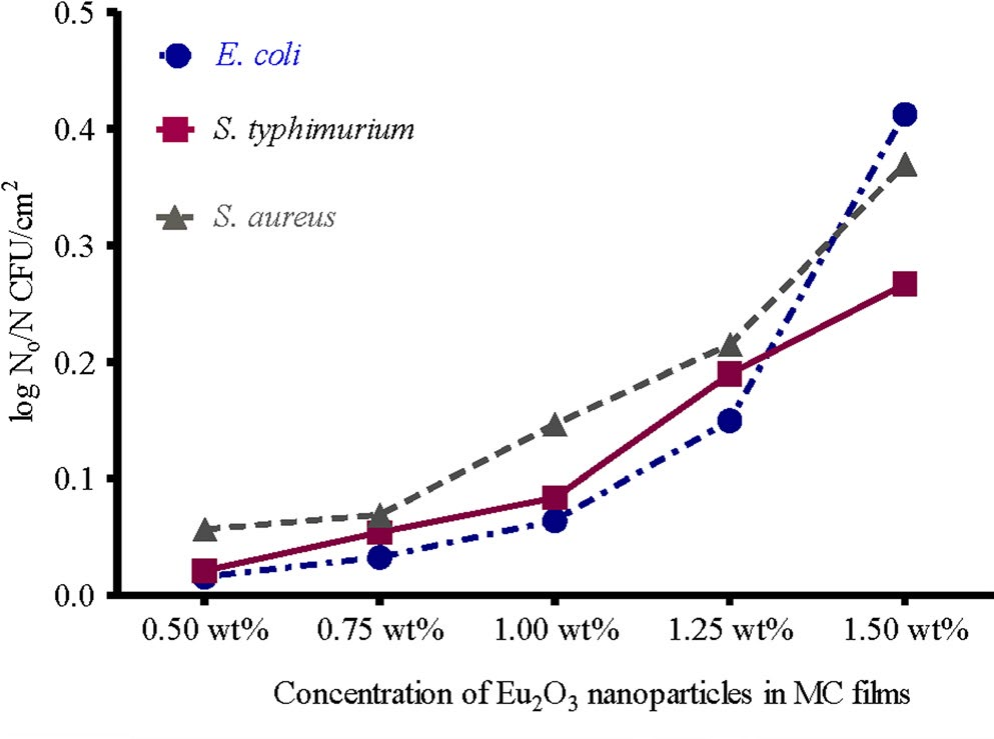
Logarithmic viability reduction (log *N*_0_/*N*) test strains in MC films doped with different concentration of Eu_2_O_3_ nanoparticles. (*N*_0_ = initial microbial load and *N* = microbial load after treatment)

The obtained reductions were ranged from 3.3 to 4.54 log cfu/cm^2^ for *E. coli*, 3.42 to 4.42 log cfu/cm^2^ for *S. typhimurium* and 3.52 to 4.19 log cfu/cm^2^ for *S. aureus* (Figure 3). These results were statistically significant (*p* < .05). Moreover, *E. coli* test strains had the highest log reduction compared with the other test strains (Figure 4). (Tunç & Duman, 2011) investigated the effects of methyl cellulose/carvacrol/montmorillonite nanocomposite films for the growth of *E. coli* and *S. aureus* on sausage. They recorded a small log reduction values of 0.90 and 0.65 log cfu/ml for E. coli, 0.90 and 0.70 log cfu/ml for *S. aureus*, at the end of 21 days of food samples storage. Their study depends on the release of antimicrobial agent (carvacrol) from films which was affected by the storage temperature of food samples. While in this study, the inoculated MC/Eu2O3 nanocomposite films were kept at room temperature for 2 hr with direct contact with the test strains.

It was assumed that gram‐negative bacteria are more reactive to environmental modifications than gram‐positive cells (Shigehisa, Ohmori, Saito, Taji, & Hayashi, 1991).

**Figure 5:**
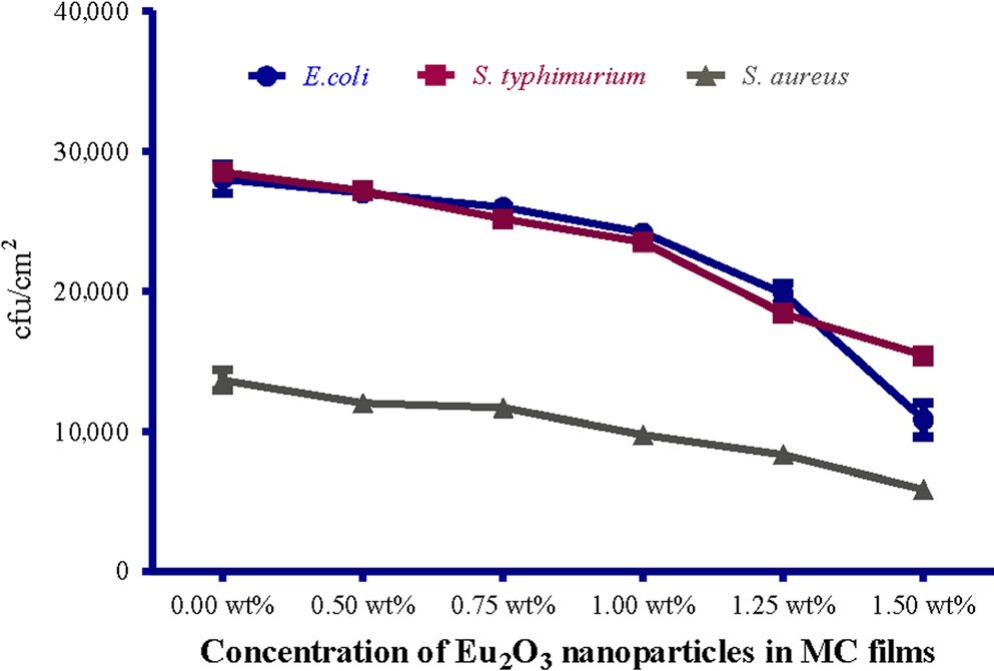
Initial adherence assays of test strains on MC films doped with different concentration of Eu_2_O_3_ nanoparticles

**Figure 6:**
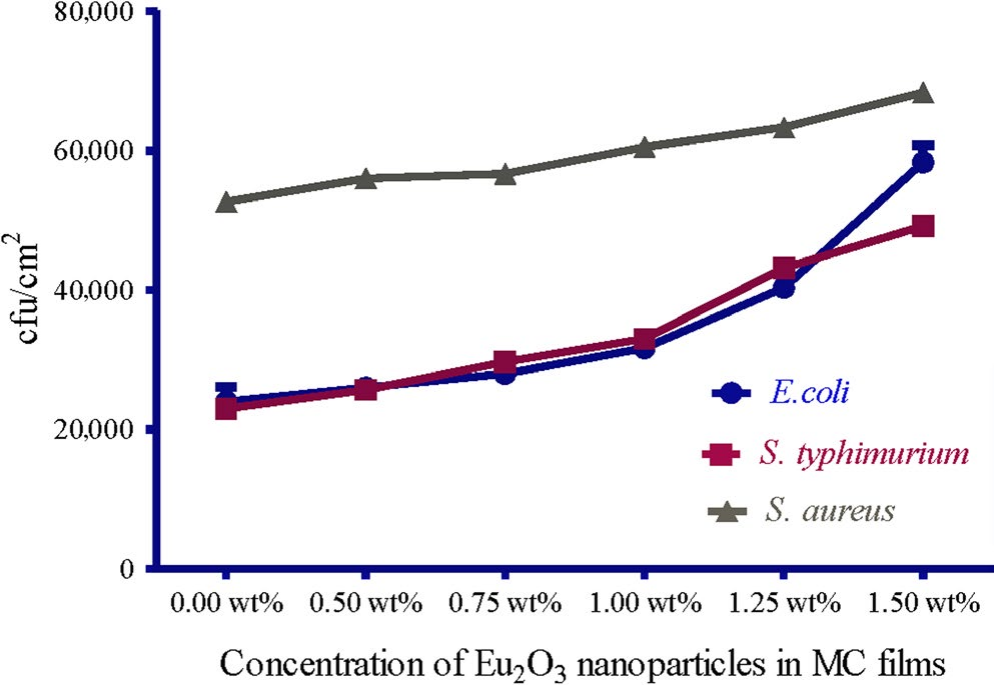
The weak attachment of test strains on Methyl Cellulose films doped with different concentration of Eu_2_O_3_ nanoparticles

The initial adherence of the test strains to MC films was studied. The adherence ability of the test strains to MC films was reduced significantly (*p* < .05) in MC films doped with different concentration of Eu_2_O_3_ nanoparticles compared with pure MC film. Moreover, MC film doped with 1.5 wt% Eu_2_O_3_ nanoparticles was the most effective concentration that could reduce the adherence of test strains and these findings confirmed what has been achieved in this study (Figure 5). Another confirmatory method to emphasize our obtained data in this study was done through testing the week attachment of the test strains against the prepared MC films. The number of recovered bacterial cells after 30 min of attachment to MC films followed by three times rinsing in phosphate‐buffered saline were recorded (Figure 6). *S. aureus* strains exhibited weak attachment to MC films compared with other test strains and this was confirmed by the large number of *S. aureus* strains compared to other strains recovered from the homogenate (Figure 6).

The obtained results showed that *S. aureus* strains were less adhere to MC films surface compared with other test strains and these findings could be attributed to the nonmotile feature of *S. aureus* (nonflagellate cocci) compare with the other flagellate test strains. Flagella are clearly implicated in the attachment of bacteria (Notermans & Kampelmacher, 1974). Bacterial flagella endow the organism with motility and the ability to respond to a chemotactic stimulus (Lillard, 1985). *S. aureus* cells showed more hydrophobic features compared with *E. coli* that were found to be moderately hydrophilic (Burks et al., 2003; Mitik‐Dineva et al., 2009). *S. aureus* cells have a hydrophobic nature which due to the extreme negative charge and the existence of hydrophobic teichoic and lipoteichoic acid in their cell wall (Canepari, Boaretti, Lleo, & Satta, 1990; Gross, Cramton, Götz, & Peschel, 2001).

Our findings reported the potential antimicrobial activity of MC films doped with Eu_2_O_3_ nanoparticles. The concentration of the Eu_2_O_3_ nanoparticles required to perform activity against foodborne microorganisms is important to use it effectively in food package. A reduction with 5 log in viability is important to attain for better foodborne pathogen reduction (Food & Administration, 2004; Olaimat & Holley, 2012). However, MC films doped with 1.5 wt% Eu_2_O_3_ nanoparticles showed a greater effect to reduce foodborne pathogen from food product's surface.

In literature, the antimicrobial activity of both MC homoplymer (de Dicastillo, Bustos, Guarda, & Galotto, 2016; Tunç & Duman, 2011) and Eu_2_O_3_ nanoparticles (Iconaru, Motelica‐Heino, & Predoi, 2013) was studied of each.

**Figure 7:**
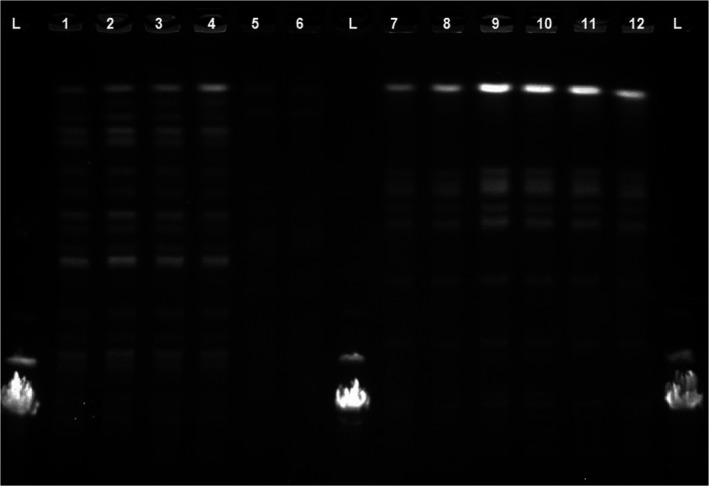
The clonal relatedness of the test strains after inoculation on MC films by using PFGE. Isolated *E. coli* from MC films doped with different concentration of Eu_2_O_3_ nanoparticles ([1] 0.00 wt%, [2] 0.50 wt%, [3] 0.75 wt%, [4] 1.00 wt%, [5] 1.25 wt%, and [6] 1.50 wt%). Isolated *Salmonella* from MC films doped with different concentration of Eu_2_O_3_ nanoparticles ([7] 0.00 wt%, [8] 0.50 wt%, [9] 0.75 wt%, [10] 1.00 wt%, [11] 1.25 wt%, and [12] 1.50 wt%)

**Figure 8:**
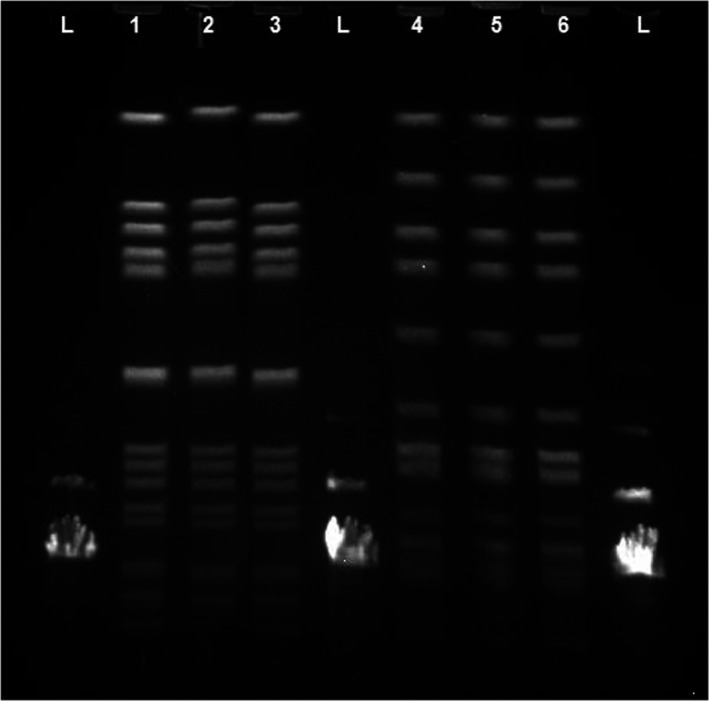
The clonal relatedness of the test strains after inoculation on MC films by using PFGE. Isolated *S. aureus* from MC films doped with different concentration of Eu_2_O_3_ nanoparticles ([1] 0.00 wt%, [2] 0.50 wt%, [3] 0.75 wt%, [4] 1.00 wt%, [5] 1.25 wt%, and [6] 1.50 wt%)

The clonal relatedness of the test strains was studied after inoculation on MC/Eu_2_O_3_ nanocomposite films using PFGE. The achieved findings showed that the clonal relatedness of test strains have not been influenced after inoculation on MC/Eu_2_O_3_ nanocomposite films (Figures 7 and 8).

## 4 CONCLUSION

Antimicrobial packaging is a better idea for food packaging with a great interest by researchers. Various MC nanocomposite films were developed to be used for safe food free from foodborne pathogens contamination. The results obtained from XRD analysis reveals the semicrystalline structure of the MC films and confirm the nanocomposite structures of the films obtained. Addition of Eu_2_O_3_ nanoparticles to the MC matrix led to a decrease in the count of inoculated *E. coli, S. typhimurium* and *S. aureus* strains (*p* ≤ .05), with reduction of 3.3 to 4.54 log cfu/cm^2^ for *E. coli*, 3.42 to 4.42 log cfu/cm^2^ for *S. typhimurium* and 3.52 to 4.19 log cfu/cm^2^ for *S. aureus*. The adherence ability of the test strains to MC/Eu_2_O_3_ nanocomposite films was reduced significantly (*p* < .05). MC films doped with 1.50 wt% Eu_2_O_3_ nanoparticles exhibited the strongest activity against the test strains. Moreover, MC film doped with 1.5 wt% Eu_2_O_3_ nanoparticles was the most effective concentration. The prepared MC/Eu_2_O_3_ nanocomposite films could be used as active food packaging materials in the food industry.

## CONFLICT OF INTEREST

All authors declare that there is no conflict of interest.

## ETHICAL APPROVAL

This study does not involve any human or animal testing; this study was approved by the Review Board of the Microbiology laboratory of Molecular Diagnostic and Personalised Therapeutics unit (MDXPTU), Hail University; this study conforms to the Declaration of Helsinki, US.
